# Correction to: ‘Diet changes thermal acclimation capacity, but not acclimation rate, in a marine ectotherm (*Girella nigricans*) during warming’ (2023), by Hardison *et al*.

**DOI:** 10.1098/rspb.2024.2055

**Published:** 2024-10-02

**Authors:** Emily A. Hardison, Gail D. Schwieterman, Erika J. Eliason

**Affiliations:** ^1^ University of California Santa Barbara, Santa Barbara, California, USA; ^2^ School of Marine Sciences, University of Maine, Orono, ME 04469, USA; ^3^ University of California, Santa Barbara, CA 93106, USA

Hardison EA, Schwieterman GD, Eliason EJ. 2023. Diet changes thermal acclimation capacity, but not acclimation rate, in a marine ectotherm (*Girella nigricans*) during warming. Proc. R. Soc. B Biol. Sci. 290, 20222505. https://doi.org/10.1098/rspb.2022.2505

There was an error in the code used to generate plot *d* in figure 5, such that crosses (X) overlayed on the correlation plot were incorrect. However, the correlation values in the plot were calculated correctly. The code has been fixed in the online repository, and the corrected version of figure 5*d* is included below. No other changes are needed to the content of the manuscript, as the error does not affect the conclusions of the manuscript and does not require any changes to the written text. Additionally, there is a spelling mistake in the abstract, where ‘homoviscous’ should be changed to ‘homeoviscous’.

We apologize for any inconvenience resulting from this error.


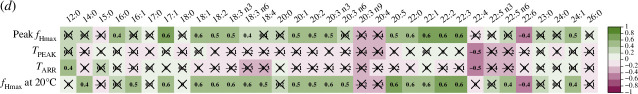


## Data Availability

This article has no additional data.

